# A Model for Apoptotic-Cell-Mediated Adaptive Immune Evasion *via* CD80–CTLA-4 Signaling

**DOI:** 10.3389/fphar.2019.00562

**Published:** 2019-05-31

**Authors:** Abraam M. Yakoub, Stefan Schülke

**Affiliations:** ^1^ Department of Molecular and Cellular Physiology, School of Medicine, Stanford University, Stanford, CA, United States; ^2^ Vice President’s Research Group: Molecular Allergology, Paul-Ehrlich-Institut, Langen, Germany

**Keywords:** apoptosis, immunotolerance, autoimmunity, costimulatory pathway, CD80, CTLA-4, coinhibitory pathway, immune evasion

## Abstract

Apoptotic cells carry a plethora of self-antigens but they suppress eliciting of innate and adaptive immune responses to them. How apoptotic cells evade and subvert adaptive immune responses has been elusive. Here, we propose a novel model to understand how apoptotic cells regulate T cell activation in different contexts, leading mostly to tolerogenic responses, mainly *via* taking control of the CD80–CTLA-4 coinhibitory signal delivered to T cells. This model may facilitate understanding of the molecular mechanisms of autoimmune diseases associated with dysregulation of apoptosis or apoptotic cell clearance, and it highlights potential therapeutic targets or strategies for treatment of multiple immunological disorders.

## Apoptosis

Apoptosis, or programmed cell death (PCD), is the physiological form of cell death that plays an important role in tissue homeostasis and regeneration, as well as maintenance of robust organ functions. While cell death by necrosis may have immunostimulatory and inflammatory effects (Sauter et al., 2000), apoptosis shows no immunostimulatory capacities, and may serve beneficial functions to the host (Sauter et al., 2000; Mahajan et al., 2016).

Our understanding of many aspects of apoptosis has constantly increased over the past ~30 years, due to tremendous effort and a myriad of studies using various model systems. For example, mouse models, due to the power of mouse genetics and with the availability of gene-targeting approaches, have been a mainstay tool to understand both the various functions of apoptosis-related genes in development and the association between gene functions or apoptosis states and disease phenotypes in mammals (see, for instance, Reyes et al., 2010; Gómez-Sintes et al., 2011; Yamaguchi et al., 2011; Wu et al., 2015). Given that the apoptosis machinery is evolutionarily conserved, from worms to mammals, other eukaryotic models have also been used such as drosophila, especially considering the ease of genetic screens and availability of a large number of fly lines (see, for instance, Richardson and Kumar, 2002; Gullaud et al., 2003; Denton et al., 2008; Xu et al., 2009). Even yeast, which, while it lacks main regulators of mammalian apoptosis such as caspases and the B cell lymphoma 2 (Bcl-2) family members, was used to study apoptosis *via* heterologous expression of many such genes, given the advantages of the yeast model (e.g., easy manipulation *via* molecular biology or genetics, low cost, and the availability of powerful tools such as the yeast two-hybrid system) (Fleury et al., 2002; Kazemzadeh et al., 2012). In addition to the *in vivo* models, *in vitro* models have also been frequently used to understand the signaling pathways or molecular interactions that regulate apoptosis at the cellular level, in physiological or disease conditions (Calissano et al., 2009; Spencer and Sorger, 2011). Importantly, with the advent of stem cell technologies and *in vitro* differentiation methods, many human (stem cell-derived) cell types, including neurons, were used to understand apoptosis-related molecular disease mechanisms in the human genetic background (Csöbönyeiová et al., 2016; Fang et al., 2018).

Induction of cell death by apoptosis in mammals is initiated by two major signaling cascades: the “extrinsic” and “intrinsic” pathways of apoptosis (Nagata and Tanaka, 2017). In the intrinsic pathway, activation of apoptosis is triggered by either developmental signals or genotoxic substances resulting in the release of many proteins including cytochrome C from the mitochondria by pro-apoptotic members of the Bcl-2 family (Nagata and Tanaka, 2017). The released cytochrome C subsequently mediates the formation of apoptosomes in the respective cell’s cytosol, which are multiprotein complexes consisting of cytochrome C, pro-caspase 9, and apoptotic protease-activating factor 1 (APAF1) that process pro-caspase 9 to its mature form (Liu et al., 1996; Zou et al., 1997, 1999). Mature caspase 9 finally mediates the maturation of inactive pro-caspase 3 to its active form caspase 3 (Nagata and Tanaka, 2017). In the extrinsic pathway of apoptosis, binding of FasL (Fas Ligand, expressed on the surface of the apoptosis-inducing cell) to Fas (CD95, tumor necrosis factor receptor superfamily member 6) on the cell destined to undergo apoptosis results in a conformational change in the Fas trimer allowing for the formation of the death-inducing signaling complex (DISC) (Nagata and Tanaka, 2017). DISC is a multiprotein complex containing the Fas-associated death domain protein (FADD) and pro-caspase 8 (Chinnaiyan et al., 1995; Kischkel et al., 1995; Muzio et al., 1996). DISC activation results in the production of mature caspase 3 by DISC-matured caspase 8 (Nagata and Tanaka, 2017). Finally, caspase 3 activated by both apoptosis pathways triggers the apoptosis program *via* the cleavage of >500 cellular substrates (Nagata and Tanaka, 2017). While FasL expression is restricted to cytotoxic T lymphocytes, T helper type-2 (Th2) cells, and Natural Killer (NK) cells (Kägi et al., 1994; Lowin et al., 1994), Fas is expressed by most cell types (Nagata and Tanaka, 2017). Therefore, FasL-Fas interaction-induced apoptosis is very important for tissue homeostasis. Besides FasL, other ligands such as tumor necrosis factor-alpha (TNF-α), lymphotoxin-alpha (LT-α), TNF-like protein-1A (TL1A), and Apo2L/TNF-related apoptosis-inducing ligand (TRAIL) can also trigger Fas-dependent apoptosis *via* the extrinsic pathway (Yamada et al., 2017).

## Apoptotic Cells and Innate Immunity

It was initially thought that apoptotic cells (ACs) might be immunologically null, however a plethora of evidence has since then indicated that ACs are immunologically active, exerting, in most cases, anti-inflammatory and immunosuppressive effects. Early, in 1997, a pioneering study (Voll et al., 1997a) showed that peripheral blood-derived macrophages exposed to ACs exhibited enhanced production of the immunosuppressive cytokine interleukin (IL)-10, which is an important immune regulatory molecule that prevents inflammatory immune responses, tissue damage, and the development of autoimmunity. Recently, ACs were shown to induce upregulation of the transcription factor aryl hydrocarbon receptor (AhR) in a Toll-like receptor (TLR) 9-dependent manner, which enhanced production of IL-10 to mediate AC-dependent immunosuppression (Shinde et al., 2018). Consequently, AhR knockout induced autoimmune responses and systemic lupus erythematosus (SLE) disease in a mouse model (Shinde et al., 2018). However, it is important to note that, while IL-10 is mainly considered to have anti-inflammatory effects on a wide range of target cells, recent findings suggest a more complex modulatory function of this important cytokine. Because of its role as an important B cell growth and differentiation factor (that promotes B cell proliferation and IgG production), IL-10 was suggested to contribute to the pathology of SLE *via* activation of autoreactive B cells (reviewed in Geginat et al., 2016). IL-10 levels were shown to increase in SLE patients and polymorphisms in the IL-10 promoter were strongly associated with SLE development (Peng et al., 2013). In line with these findings, neutralization of IL-10 blocked autoantibody production in SLE patients (Llorente et al., 1995). However, both the source of the pathogenic IL-10 production in SLE patients and its possible contribution to other autoimmune diseases remain to be further characterized (Geginat et al., 2016).

Besides IL-10, ACs were shown to induce the production of many anti-inflammatory cytokines such as transforming growth factor beta (TGF-β), platelet activating factor (PAF), and prostaglandin E2 (PGE2) (Voll et al., 1997b; Cvetanovic and Ucker, 2004). In addition, macrophage exposure to ACs caused a reduction in the macrophages’ expression of the pro-inflammatory and immunostimulatory cytokines tumor necrosis factor (TNF)-α, IL-12, IL-1β, IL-18, and granulocyte-macrophage colony-stimulating factor (GM-CSF) (Fadok et al., 1998; Kim et al., 2005).

Therefore, ACs are able to modulate the activation state of, and cytokine secretion from, antigen-presenting cells (APCs) which influences both innate and subsequent adaptive immune responses to the ACs. This immune modulation also has consequences for T cell activation upon encountering ACs. For example, suppression of macrophage-derived IL-12 production may prevent the differentiation of self-reactive T helper type-1 (Th1) CD4^+^ cells and autoimmunity (Trembleau et al., 1995), while AC-induced IL-10 represses the expression of MHC-II and costimulatory molecules required for antigen presentation and subsequent T cell activation (Couper et al., 2008).

## Apoptotic Cells and Adaptive Immunity

### The Route to T Cell Activation

Upon T cell receptor (TCR) activation by ligand binding, such as by specific-antigen-bound major histocompatibility complex (MHC) on the surface of APCs, the TCR-associated CD3 chains become tyrosine-phosphorylated leading to recruitment of kinases and scaffold proteins and formation of a supramolecular complex that triggers signaling pathways and transcriptional cascades responsible for T cell differentiation and clonal expansion, as well as effector cell generation (Rathmell et al., 2003; Smith-Garvin et al., 2009; Marko et al., 2010; Schultze et al., 2012). Among those signaling and transcriptional events are the upregulation of the glucose receptor Glut 1 (Frauwirth et al., 2002) and the glutamine receptors Snat1 and Snat2 (Carr et al., 2010), and the activation of the MAPK and PI3K/Akt pathways (Kannan et al., 2012). Collectively, these events are required to fulfill the metabolic needs of the proliferating T cells and to support cell cycle progression and cytokine production (Appleman et al., 2002).

However, TCR ligation alone is insufficient to trigger or maintain robust T cell activation, as this process is tightly regulated by a complex array of costimulatory and coinhibitory ligands and receptors (Esensten et al., 2016). The prototypical costimulatory receptor is CD28, while the prototypical coinhibitory receptors are cytotoxic T lymphocyte antigen-4 (CTLA-4) and PD-1 (Buchbinder and Desai, 2016). Shared ligands between both types of receptors include CD80 (B7-1) and CD86 (B7-2). Stimulation of CD28 potentiates and sustains IL-2 production from T cells and prevents peripheral immunotolerance development (Bour-Jordan et al., 2011; Kow and Mak, 2013). These T cells activated by CD28-B7 signaling then mature and differentiate, subsequently inducing B cell proliferation and differentiation into plasma cells producing antigen-specific antibodies (Kow and Mak, 2013).

Although CD80 can bind to and activate CD28, significant evidence suggests that it also contributes a strong coinhibitory function. In fact, the binding of CD80 to the coinhibitory receptor CTLA-4 occurs with higher affinity than its binding to the costimulatory receptor CD28 (*K*_D_ = 0.2 and 4 μM, respectively) (van der Merwe and Davis, 2003; Collins et al., 2005; Butte et al., 2008). Furthermore, the crystal structure of the CD80-CTLA-4 complex showed that CD80 homodimers bind bivalent CTLA-4 homodimers in an unusually stable, high-avidity complex (Ikemizu et al., 2000; Stamper et al., 2001). CTLA-4-mediated coinhibitory signaling is critical for negative regulation of T cell activation and proliferation, as evidenced by the severe lymphoproliferation and multi-organ inflammatory lymphocytic infiltrates observed in mice lacking CTLA-4 signaling (Tivol et al., 1995) or in cancer patients receiving anti-CTLA-4 antibodies (Pardoll, 2012).

Even when the TCR and CD28 are ligand-activated, CTLA-4 activation can inhibit cell cycle progression and cause proliferative arrest of T cells by suppressing IL-2 production (Krummel and Allison, 1996; Walunas et al., 1996). It thus seems that CTLA-4 has a superdominant, overarching role in the regulation of T cell activation. CTLA-4 selectively reverses CD28-mediated costimulation (Walunas et al., 1996; Tai et al., 2007). Moreover, since CTLA-4 is a higher affinity CD80/86 binding partner, CTLA-4 can compete out CD28 for CD80/CD86 binding, also leading to suppression of T cell activation. Besides CD28 and CTLA-4, other costimulatory and coinhibitory receptors and ligands were later discovered and reported to modulate APC-T cell interaction (for review, see Kow and Mak, 2013; Attanasio and Wherry, 2016; Schildberg et al., 2016).

### An Integrative Model of Apoptotic-Cell-Mediated Immune Evasion and Tolerance

Strong evidence indicates an essential role for the coinhibitory pathway in suppressing adaptive immune responses. First, the role of the coinhibitory signaling in regulating self-tolerance or autoimmunity is supported by the finding that various coinhibitory ligands are expressed, besides APCs, on non-hematopoietic cells which was suggested to play a role in maintaining tissue tolerance by suppressing self-reactive T cells in the periphery (Anderson et al., 2016; Schildberg et al., 2016; Ward-Kavanagh et al., 2016; Janakiram et al., 2017). Notably, some tumors and infectious pathogens evade immune recognition by exploiting such natural tolerance mechanisms (Odorizzi and Wherry, 2012; Pardoll, 2012; Wang et al., 2013; Attanasio and Wherry, 2016; Baumeister et al., 2016).

Moreover, CTLA-4-mediated coinhibition was shown to be essential for terminating T cell activation as CTLA-4^−/−^ mice develop massive lymphoproliferation and early death (Tivol et al., 1995; Waterhouse et al., 1995). CTLA-4 was also suggested as a master switch for peripheral tolerance (Bluestone, 1997). Importantly, the essential role of CTLA-4 in autoimmune regulation was further highlighted by the fact that blockade of CTLA-4 signaling in multiple animal models resulted in aggravation of autoimmune diseases (Karandikar et al., 1996; Luhder et al., 1998; Chitnis et al., 2001). Moreover, CTLA-4 gene single-nucleotide polymorphisms (SNPs) in humans were associated with autoimmune disorders. For example, a SNP in the 6.1-kb (kilobase) 3′ region of the CTLA-4 gene was associated with higher risk of Grave’s disease, autoimmune hypothyroidism, and type 1 diabetes mellitus (Ueda et al., 2003). Blockade of the CTLA-4- or PD-1-mediated coinhibitory signaling accelerated cardiac allograft rejection in C57BL/6 mice receiving BALB/c hearts (Ito et al., 2005). It was suggested that CTLA-4 and PD-1 may play redundant or complementary functions that differentially target different stages of tolerance (priming, activation, or reactivation of T cells, respectively) (Linsley and Ledbetter, 1993; June et al., 1994; Dahl et al., 2000).

The role of the costimulatory/coinhibitory molecules in immune responses to ACs *in vivo* (in mice) has also been suggested by the finding that antigen-coupled ACs (derived from splenocytes) induced T cell tolerance *via* enhanced IL-10 and PD-L1 expression on AC-ingesting macrophages. These ACs also enhanced Treg activation that maintained immunotolerance in that model (Kushwah et al., 2010). PD-L1 upregulation was dependent on IL-10, as IL-10 neutralization with antibodies reduced the PD-L1 response to ACs.

Since ACs were reported to induce IL-10, and given that IL-10 is mostly an anti-inflammatory cytokine as discussed above, which is important for the induction of tolerance and suppression of dendritic cell maturation (Faulkner et al., 2000; Corinti et al., 2001), it seemed plausible to suggest that adaptive immune responses to ACs could be mediated by IL-10 effects. However, that hypothesis has been difficult to fully establish due to different conclusions from various studies. For example, ACs were shown to induce IL-10 production by monocytes (Voll et al., 1997a), but not macrophages (Fadok et al., 1998). But antigen-coupled ACs enhanced IL-10 and PD-L1 expression in AC-ingesting macrophages and induced T cell tolerance in mice (Getts et al., 2011). Importantly, however, while statistically significant, the IL-10-induced PD-L1 upregulation in that study was subtle when compared to the IL-10-neutralized control (only ~20% increase in PD-L1 mean fluorescence intensity (MFI) over the control). Conversely, using macrophage cell lines and mouse primary macrophages, we found that the upregulation of another costimulatory/coinhibitory molecule, CD80, was more pronounced (on average > 4-fold upregulation, relative to the unstimulated control) (Yakoub et al., 2018).

In another study, dendritic cells exposed to ACs exhibited reduced T cell proliferation and activation and reduced lipopolysaccharide (LPS)-triggered upregulation of the costimulatory molecule CD86 (Stuart et al., 2002). In that study, ACs did not significantly affect IL-10 levels secreted by dendritic cells, and even IL-10 neutralization by soluble IL-10R did not affect expression of the costimulatory molecules on the dendritic cells (Stuart et al., 2002). Even further, ACs could inhibit LPS-induced activation of bone marrow-derived dendritic cells derived from IL-10-deficient mice; and neutralizing another anti-inflammatory cytokine, TGF-β1, could not suppress the inhibition of dendritic cell maturation by ACs (Stuart et al., 2002), contrary to what was suggested (Chen et al., 2001). In total, these reports suggest that the effect of ACs on adaptive immune responses cannot, or cannot completely, be attributed to secondary effects of the cytokine secretion modulated by ACs, and that ACs may have direct effects on the machinery regulating adaptive immune responses.

Similar to the results in macrophages showing upregulation of CD80 by ACs (Yakoub et al., 2018), ACs were reported to induce CD80 and CD86 expression on *in vitro* differentiated human dendritic cells, which involved both soluble factors secreted and cell-cell contact to achieve the full effects of the ACs (Johansson et al., 2007; Pathak et al., 2012). Notably, however, these effects of ACs on CD80/86 expression on dendritic cells required “pre-activation” of the ACs with anti-CD3 and anti-CD28 antibodies, whereas non-pre-activated ACs showed no effect on CD80/86 expression on dendritic cells (Johansson et al., 2007; Pathak et al., 2012). These results suggest a differential AC response between dendritic cells and macrophages, as such AC activation was not required to modulate CD80 levels on macrophages (Yakoub et al., 2018). In a similar concept, “heat-stressed,” but not unstressed, ACs induced the costimulatory molecules CD40, CD80, and CD86 on dendritic cells and secretion of the immunostimulatory IL-12, resulting in enhanced T cell responses (Feng et al., 2002).

**Figure 1:**
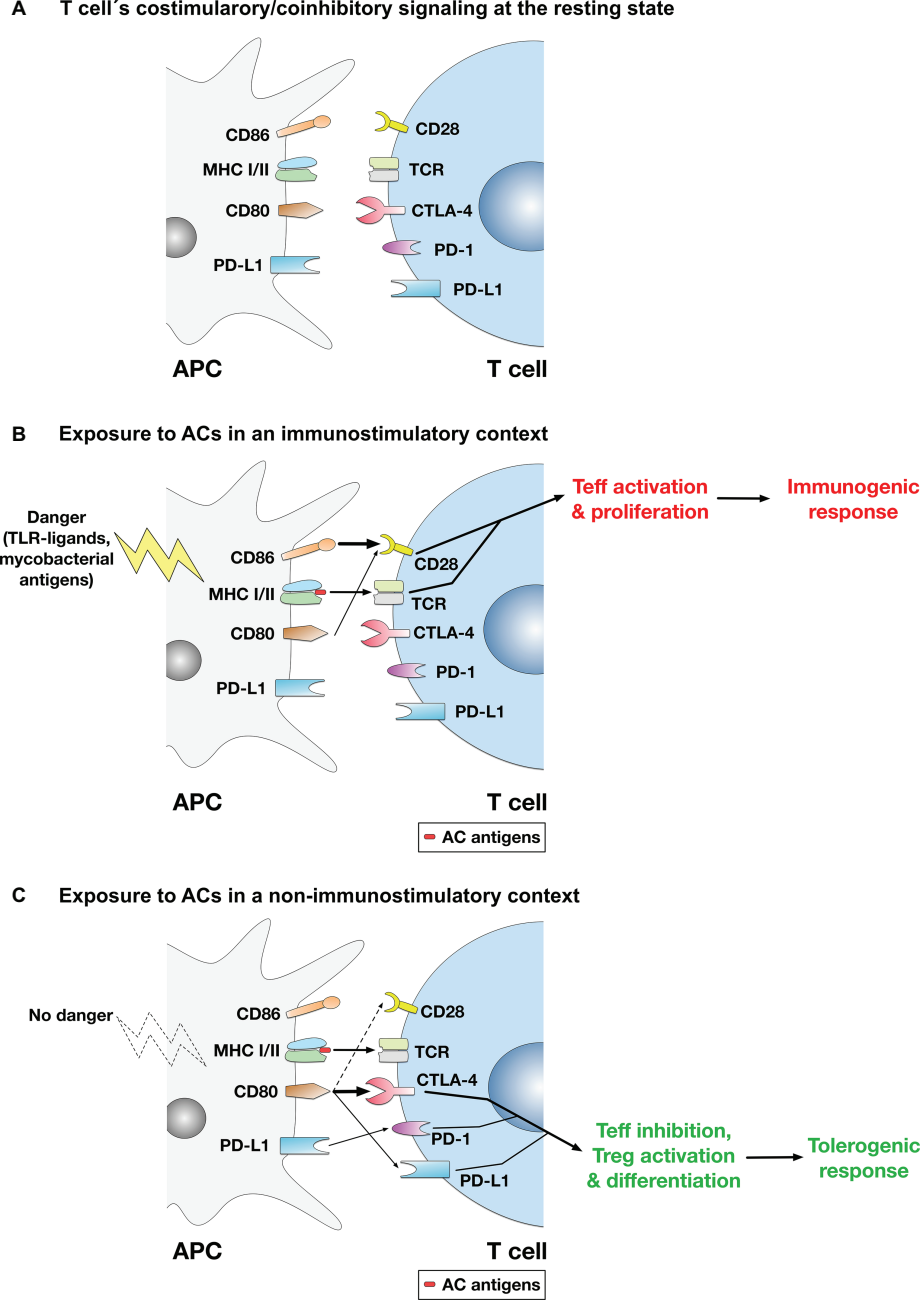
A model for the regulation of T cell-mediated adaptive immune responses to apoptotic cells. **(A)** At the resting state, no activation of the TCR or the costimulatory pathway takes place. **(B)** In immunostimulatory contexts (such as AC ingestion by APCs in the presence of LPS, TLR ligands, or mycobacterial antigens), TCR binding to AC antigens presented in the context of MHC-I/II and the costimulatory signaling mediated by binding of CD28 by mainly CD86 (or CD86 and CD80) take place. Thus, activation of the costimulatory pathway ensures effector T cell (Teff) activation and proliferation, leading to mounting of an immune response to AC antigens. **(C)** In non-immunostimulatory contexts, such as AC ingestion by APCs in the absence of additional immunopotentiating stimuli (e.g., TLR ligands or mycobacterial antigens), the costimulatory signaling mediated by CD28 is downregulated. Rather, CD80 is upregulated, which binds mainly to CTLA-4 (CD80 binds CTLA-4 with much higher affinity than CD28), initiating the coinhibitory signaling that leads to Teff suppression and apoptosis. Even if some costimulatory signal is conveyed (by binding of some CD80 molecules to CD28), the CTLA-4-mediated coinhibitory signal predominates and usually overrides costimulation. Other signals are possible (e.g., binding of CD80 to PD-L1 on T cells or binding of PD-L1 on APCs to PD-1 on T cells) and might have some contribution to the overall coinhibitory signaling delivered to T cells, although their significance and relative contribution have yet to be established. Overall, the coinhibitory signaling to T cells triggers the inhibition of Teff functions; and in this context, differentiation of Tregs may also be enhanced.

While the immunosuppressive or tolerogenic effects of ACs are established by a plethora of evidence (Kabelitz and Janssen, 1997; Steinman et al., 2000; Fadok et al., 2001; Ferguson et al., 2002; Liu et al., 2002; Stuart et al., 2002; Morelli et al., 2003; Rovere-Querini et al., 2004; Gray et al., 2007), there were also instances where ACs were reported to have immunostimulatory effects (Hoffmann et al., 2000; Ignatius et al., 2000; Feng et al., 2002; Buttiglieri et al., 2003; Goldszmid et al., 2003; Ishii et al., 2003; Casares et al., 2005). It is thus possible to propose a model (Figure 1) whereby exposure to ACs in a non-inflammatory/non-immunostimulatory context (that does not involve immunogenic stimuli that trigger T cell activation) mounts a tolerogenic response *via* T cell inhibition through the coinhibitory pathway, whereas exposure to activated ACs in an immunostimulatory context mounts an immunogenic response *via* T cell activation through the costimulatory pathway. In support of this model is our finding that under non-immunostimulatory conditions, the AC-induced CD80 upregulation on macrophages was coupled with CD28 downregulation on T cells (Yakoub et al., 2018), which may possibly enhance the coinhibitory functions of CD80 as it binds the coinhibitory receptor CTLA-4 with much higher affinity and stability than it does the costimulatory receptor CD28 as described above.

CD80 binding to CTLA-4 conveys an inhibitory signal to T cell activation that overrides costimulatory signals, counteracting the initiation of T cell activation and proliferation and indeed inducing their apoptosis (Walunas et al., 1994). Moreover, other mechanisms could also contribute to AC-mediated suppression of adaptive immune responses. For example, ACs (derived from dendritic cells) engulfed by dendritic cells induced TGF-β1 secretion and differentiation of naïve T cells into Foxp3^+^ Tregs (Kushwah et al., 2010). Naïve T cells can differentiate upon antigen recognition into effector T cell subsets such as Th1, Th2, and Th17, or into immunosuppressive Tregs. AC-ingesting dendritic cells suppressed the development of the effector Th17 cells, but enhanced the development of Tregs where dendritic cell-T cell interaction and the costimulatory/coinhibitory signaling were suggested to play a role in Treg induction (Yamazaki et al., 2003; Torchinsky et al., 2009).

CD80 expressed on T cells was also shown to bind PD-L1 on APCs with an affinity greater than that of CD80-CD28 binding (Butte et al., 2007; Rollins and Gibbons Johnson, 2017), which may negatively regulate T cell activation. Similarly, CD80 on APCs, which ACs upregulate (Yakoub et al., 2018), was suggested to bind PD-L1 on T cells (Schildberg et al., 2016), which may downregulate T cell activation, if not directly, indirectly by competing out CD28 and thus reducing the costimulatory signal that is essential for T cell activation and sustenance of the adaptive immune response. Notably, PD-L1 expression on parenchymal tissues including pancreatic islets mediated tolerance and inhibited self-reactive CD4 T cells (Keir et al., 2006); and interference with CD80-PD-L1 binding enhanced activation of CD4 and CD8 T cells *in vivo* and accelerated development of autoimmune diabetes in NOD mice (Paterson et al., 2011). Interestingly, it was also proposed that CD80/86 binding to CTLA-4 and PD-L1 on T cells enhances T cell motility, reducing T cell-APC contacts and the strength of the immune synapse, while enhancing contacts with, and activation of, Tregs (Dilek et al., 2013). Moreover, binding of PD-L1 on ACs to PD-1 on T cells is also possible (Kushwah et al., 2010), which may also strengthen the coinhibitory signal to T cells, although its significance needs to be established as previously discussed.

Whether ACs would induce a tolerogenic or immunogenic response may depend on presence of immunostimulatory conditions or cues, including: (1) the type of cell death induced in the ACs, e.g., caspase-dependent or independent (Larmonier et al., 2002); (2) type of the apoptosis-inducing agent or drug used in the experimental model (Casares et al., 2005; Obeid et al., 2007); (3) the stage of apoptosis (early vs. late) in the ACs (Weyd et al., 2013); (4) type of the apoptotic corpse (ACs or apoptotic blebs) ingested by the APC (Fransen et al., 2009); (5) cell-type of the ACs ingested by the APCs (Kushwah et al., 2010); (6) secretion of T cell immuostimulatory cytokines, such as TNF-α, or TLR ligands (Clayton et al., 2003); (7) type and ratio of the APCs (dendritic cells or macrophages) present in a particular tissue site (Denning et al., 2007); or (8) presence of potentially immunogenic or immunogenizing infectious pathogen (e.g., mycobacterial) antigens in the tissue microenvironment (Espinosa-Cueto et al., 2017).

While the distinction between APC types (dendritic cells or macrophages) in terms of the type of immune response (immunogenic vs. tolerogenic) to ACs cannot be completely settled given the varying reports thus far in this regard, there is still some preliminary evidence to propose that macrophages might mainly mediate tolerogenic responses to ACs while dendritic cells might mainly mediate the immunogenic responses. Dendritic cells and macrophages exhibit distinct locales and may thus mediate differential, locale-specific, AC responses (T cell activation or inhibition). For example, in the intestinal lamina propria, both APC types present commensal microbe and dietary antigens to T cells, with dendritic cells inducing effector Th17 T cells, and macrophages inducing Tregs (Denning et al., 2007). Tregs induced by AC-presenting macrophages showed induced anti-inflammatory cytokine production and reduced immunostimulatory cytokines, and displayed an anergic phenotype after restimulation with the antigen (Denning et al., 2007). Because macrophages are more abundant than dendritic cells in the lamina propria, T cell tolerance thus becomes the predominant response in that context. Importantly, macrophages were shown to be essential for clearing tumor ACs introduced into mice and eliciting tolerogenic responses to the ACs (Asano et al., 2011); as ablation of the spleen marginal zone macrophages in these mice diminished the immunosuppressive potential of the ACs and enabled triggering of an immune response to ACs (McGaha et al., 2011). It thus seems possible that the immunosuppressive or tolerogenic response to ACs is mainly mediated by macrophages as the APCs.

## Role of Apoptotic-Cell-Mediated Immunosuppression in Diseases

An abundance of ACs (~70 billion) is produced daily in humans and defective clearance of these ACs has been associated with diseases, primarily autoimmune conditions. Therefore, we will briefly discuss some disease conditions, where we propose that the immunosuppressive properties of ACs may be exploited for therapeutic purposes.

### Rheumatoid Arthritis

Rheumatoid Arthritis (RA) is a chronic systemic autoimmune disorder characterized by progressive inflammatory bone and joint destruction. Immunologically, the autoimmune responses are mediated by either circulating autoantibodies directed against citrullinated peptides and rheumatoid factor or complement protein C3 (Abdolmaleki et al., 2018). The destructive autoimmune responses in RA are maintained by IL-6 and TNF-α secreted by tissue macrophages triggering the MMP and RANK-ligand-supported activation of osteoclasts (An et al., 2016). In RA patients, the sustained joint inflammation is maintained by an abnormal state of aberrant cell survival caused by perpetual T cell activation resulting in stimulation and proliferation of fibroblasts which was termed “apoptosis resistance” (Malemud, 2018). In line with these observations, anti-apoptotic proteins were shown to be upregulated in RA synovial fluids (reviewed in Williams et al., 2018). Therefore, induction of apoptosis has the potential to reduce joint damage and to further modulate autoimmune responses in RA by modulating the coinhibitory signaling to T cells as previously described. Experimental apoptosis induction has therefore been considered as potential therapeutic avenue in RA, e.g., by targeting intracellular apoptotic inhibitory molecules, but thus far has not reached clinical trials (Williams et al., 2018).

### Systemic Lupus Erythematosus

SLE is a chronic systemic autoimmune disorder characterized by the presence of circulating nuclear antigens, including DNA and nucleosomes, and of autoantibodies against these nuclear antigens (Poon et al., 2014). SLE affects the skin, lungs, kidneys, and central nervous system. Impaired engulfment of ACs by phagocytes leads to accumulation of ACs in the lymph nodes and blood and in the skin after UV exposure. Impaired AC clearance eventually leads to secondary necrosis, which allows intracellular (self-) antigens, normally hidden within the AC to be exposed extracellularly, and thus recognizing these self-antigens by the immune system, causing the production of autoantibodies and autoimmunity. While mostly suggested to be caused by defective AC engulfment, super-stimulatory APCs that lead to hyperactive T cells were also suggested to recapitulate SLE in a mouse model (Zhu et al., 2005). Thus, targeting APC activity which is normally modulated by ACs in normal physiological conditions might be a successful strategy for therapy development for SLE.

### Sjögren’s Syndrome

Sjögren’s Syndrome (SS) is an autoimmune disease targeting the salivary and lacrimal glands resulting in chronic dryness of mouth and eyes (Ainola et al., 2018). In SS, apoptotic particles blebbing from apoptotic epithelial cells, possibly caused by defects in the production of sex hormones, that contain typical SS autoantigens such as hY1RNA were shown to contribute to disease pathology (Ainola et al., 2018). In line with this increase in apoptosis rates, enhanced levels of both Fas and FasL were reported in salivary gland tissues, but not in lacrimal gland tissues or peripheral blood lymphocytes of patients with SS, implicating Fas-mediated apoptosis in the destruction of salivary gland tissue (Ishimaru et al., 2001; Bolstad et al., 2003). The presence of both autoantigens and adjuvanting nucleic acids in these apoptotic particles was shown to stimulate plasmacytoid dendritic cells in salivary glands *via* TLR7 and TLR9, resulting in the activation of autoreactive T and B cells (Ainola et al., 2018). However, Ishimaru et al. reported that mice treated with an anti-murine FasL antibody (to suppress Fas-mediated apoptosis) unexpectedly showed exacerbations of the autoimmune lesions in both salivary and lacrimal glands (Ishimaru et al., 2001). Therefore, both the role of apoptosis in the pathology of SS and the therapeutic potential of ACs in the treatment of SS are poorly understood, warranting further investigations.

### Autoimmune Lymphoproliferative Syndrome

Autoimmune Lymphoproliferative Syndrome (ALPS) is an inherited autoimmune disorder characterized by spleno- and hepatomegaly, lymphadenopathy, autoimmune lesions in multiple organs, as well as autoimmune hemolytic anemia, thrombocytopenia, or leukocytopenia, caused by cell-type specific autoantibody production (Turbyville and Rao, 2010; Price et al., 2014; Yamada et al., 2017). In ALPS patients, these symptoms are caused by spontaneous mutations in the Fas, FasL, or caspase 10 genes, resulting in a defective apoptosis of antigen-activated T- and B cells in the periphery, and the impaired limitation of immune responses (Yamada et al., 2017). Since the pathology of ALPS is caused by a disruption of lymphocyte apoptosis, it is plausible to speculate that a decrease in production of ACs (which exert immunoinhibitory/anti-inflammatory effects as discussed) may contribute to the autoimmune pathology in ALPS patients. The role of ACs in ALPS pathogenesis and its possible exploitation for therapy is thus an interesting area for future research.

### Diabetes Mellitus

Type 1 diabetes mellitus can be caused by autoimmune responses to pancreatic beta-cell antigens resulting in insulin deficiency and hyperglycemia. While it was suggested that inefficient clearing of apoptotic pancreatic cells resulting in the release of damage-associated molecular patterns (DAMPs) in combination with autoantigens may contribute to the pathology of type 1 diabetes (Heimberg et al., 2001; O’Brien et al., 2006), ACs were also suggested as a tool to induce tolerance to beta-cell self-antigens. Indeed, ACs (apoptotic beta-cell infusion) could suppress beta-cell antigen-specific CD4^+^ T cell proliferation and delay the onset of diabetes in the diabetes-susceptible, autoimmune (NOD) mice (Xia et al., 2007; Marin-Gallen et al., 2010). Therefore, ACs show a promising potential for the treatment of type 1 diabetes.

### Transplantation

After transplantation, immunosuppressive drugs are given to the patients, to induce tolerance and prevent graft rejection. However, these drugs show many undesirable and potentially dangerous side effects. Thus, ACs were suggested to be used as possibly side effect-free tolerogenic tools (Morelli and Larregina, 2010). Donor’s ACs given to transplant patients may tolerize or repress the recipient’s immune responses to the transplant, prevent graft-versus-host reactions, and enhance graft survival (Kleinclauss et al., 2006; Wang et al., 2006, 2009; Bittencourt et al., 2011).

### Cancer

Anti-tumor chemotherapeutic agents lead to production of massive cytotoxicity and generation of ACs. Given the adaptive immunosuppressive and tolerogenic effects of ACs, it is plausible to hypothesize that the apoptosis induced, and the ACs produced, by cancer treatments contribute to tumor survival indirectly by dampening immune responses to cancer cell antigens carried on these ACs. Thus, functionally blocking ACs in cancer may help reduce these undesirable effects of antineoplastic agents on anti-tumor immunity. In fact, some therapeutic strategies to target the coinhibitory molecules that mediate the adaptive immune responses to ACs have been investigated for their possible beneficial effects on anti-tumor immunity (Chen, 2004; Hirano et al., 2005; Curran et al., 2010).

## Concluding Remarks

While ACs have been investigated as tolerogenic or immunosuppressive “vaccines,” understanding the molecular mechanisms of the ACs’ immunomodulating effects, especially their interaction with the costimulatory/coinhibitory pathway, may encourage attempts at targeting specific molecules that ACs exploit in mediating their effects. In general, the costimulatory/coinhibitory pathway has been explored as target for therapeutic purposes. For example, targeting of either CD28, PD-1, PD-L1, or CTLA-4 has been investigated and some therapies targeting this machinery are already in clinical use (e.g., the anti-CTLA-4 antibody Ipilimumab for the treatment of advanced metastatic melanoma, and the anti-PD-1 antibodies Pembrolizumab and Nivolumab for the treatment of advanced melanoma, advanced non-small cell lung cancer, and metastatic renal cell carcinoma) (Dilek et al., 2013).

Although the costimulatory/coinhibitory pathway has been a tempting target for disease therapy, some challenges remain and are yet to be overcome in the future to enable full harnessing of the therapeutic potential of this pathway. For example, some therapies targeting the costimulatory/coinhibitory pathway have even been discontinued in medical practice due to limited effectiveness (Smith et al., 2013), or have failed clinical trials at early stages (Khoury and Sayegh, 2004). Some proposed therapies can also pose significant risks (Frebel and Oxenius, 2013). This all reflects the fact that we have not yet reached complete and thorough understanding of the costimulation/coinhibition pathways and their intricate interactions with diseases. In conclusion, this perspective proposes a model to understand how ACs regulate the costimulatory/coinhibitory signaling pathways of regulating T cell activation in order to suppress adaptive immune responses, which may facilitate harnessing these molecular mechanisms in therapy development for various immunopathological conditions.

## Author Contributions

AY conceived and wrote the first draft of this manuscript. SS reviewed, critiqued, and revised the first draft. AY and SS rewrote and finally revised the final manuscript.

### Conflict of Interest Statement

The authors declare that the research was conducted in the absence of any commercial or financial relationships that could be construed as a potential conflict of interest.
